# Assessing fidelity of design and training of Making Every Contact Count (MECC) in a mental health inpatient setting

**DOI:** 10.1186/s12889-024-20774-5

**Published:** 2024-11-29

**Authors:** Emma Kemp, Maria Raisa Jessica Aquino, Rob Wilson, Milica Vasiljevic, Kate McBride, Craig Robson, Sally Faulkner, Mish Loraine, Jill Harland, Catherine Haighton, Angela Rodrigues

**Affiliations:** 1 School of Psychology, University of Sheffield, S1 4DP Sheffield, UK; 2 Department of Psychology, Northumbria University, Northumberland Building, Newcastle upon Tyne, NE1 8ST UK; 3 Population Health Sciences Institute, Newcastle University, Newcastle Upon Tyne, NE1 4AX UK; 4 Department of Sociology, Manchester Metropolitan University, Manchester, M15 6BX UK; 5 Department of Psychology, Durham University, Durham, DH1 3LE UK; 6 Cumbria, Northumberland, Tyne & Wear NHS Foundation Trust, St Nicholas Hospital, Newcastle upon Tyne, NE3 3XT UK; 7 Northumbria Healthcare NHS Foundation Trust, North Tyneside General Hospital, NE29 8NH North Shields, UK; 8 North East Together, Bolingbroke Street, Newcastle upon Tyne, NE6 5PH UK; 9 Department of Social Work, Education and Community Wellbeing, Northumbria University, Coach Lane Campus West, Newcastle upon Tyne, NE7 7XA UK

**Keywords:** Public mental health, Intervention fidelity, Training implementation, Documentary analysis, Behaviour change, Health services research

## Abstract

**Background:**

Making Every Contact Count (MECC) is a public health strategy which strives to enable brief interventions to be implemented through opportunistic healthy lifestyle conversations. In a mental health inpatient setting a bespoke MECC training package has been developed to encourage cascade training through a *train the trainer* model and to incorporate an additional regional health strategy A Weight Off Your Mind into Core MECC training to provide a focus on healthy weight management. This study evaluated the fidelity of design of MECC in the mental health inpatient setting and fidelity of the training package currently being cascaded across the region.

**Methods:**

Initially a documentary analysis of six documents shared through the mental health inpatient setting including MECC implementation guide, logic model, checklist and evaluation framework. Documents were analysed using the Template for Intervention Description and Replication (TIDieR) checklist and coded using the Behaviour Change Technique (BCT) Taxonomy version one (BCTTv1) and the Expert Recommendations for Implementing Change (ERIC) Taxonomy. Coding was compared against MECC guidance documents to complete the fidelity assessment. A training delivery guide, training slides and two recordings of both train the trainer and Core MECC + A Weight Off Your Mind training were analysed for behaviour change techniques (BCTs) and compared to conduct a fidelity training assessment.

**Results:**

Overall the implementation of MECC in the mental health inpatient setting had moderate fidelity to the MECC guidance, with a total of 31 BCTs identified across guidance and provider documents and a 77% adherence of provider documents to guidance. The highest level of fidelity applied to the MECC implementation guide where 86% of identified BCTs were also present. The training package showed high fidelity that both training programmes were being delivered as intended with 100% of BCT matched from training materials to training transcripts. Potential loss of fidelity through additional BCTs was present across provider documents and training transcripts.

**Conclusion:**

The implementation of MECC across the mental health inpatient setting and the training package appear to be delivered as intended therefore demonstrating good fidelity and potential benefits to public health. Future research would benefit from assessing cascade training sessions from those who have completed *train the trainer* to evaluate ongoing fidelity of training across the trust.

## Background

Making Every Contact Count (MECC) is a public health strategy designed to promote behaviour change through brief healthy lifestyle conversations between healthcare staff and service users [29]. A recent Delphi study has established an updated consensus on the definition of MECC, capturing the evolution of MECC and increased scope since its inception [23]. The MECC approach was initially developed in Yorkshire and Humber as a response to NICE guidelines designed as a public health initiative to promote health behaviour change and provide a common framework for all NHS workforce to use [22, 26]. MECC promotes behaviour change by using opportunistic delivery of healthy lifestyle information relating to health issues, there are five core elements of MECC including smoking, healthy eating, physical activity, alcohol and mental health [13]. MECC guidance documents outline key components of MECC which impact both organisations, staff and individuals [30]. MECC guidance documents support organisations in developing leadership and strategy around the approach and through provision of training and information relating to the MECC approach, enable staff with competence and increase confidence to deliver MECC through healthy lifestyle conversations, and encourage individuals to improve their health and wellbeing [30]. In addition to the core MECC definition, a broader definition has also been recognised (referred to as MECC Plus) to recognise how core MECC training has been further developed to support conversations about the wider determinants of health and wellbeing, such as social and health inequalities (e.g., debt management and housing and welfare; see Public Health England [30]).The MECC implementation guide outlines a process for implementing MECC using an eight-step approach [17] outlined in Table 1, and provides a checklist of how this can be achieved [30].

**Table 1:** Kotter’s [17] eight-step approach for implementing MECC

Step	Description
Organisational Strategy	Identifying why MECC should be used and outlines the initial goals
Senior Leadership	Encourages organisations to identify senior leadership buy-in to ensure successful implementation of MECC
Planning	Refers to who will champion the MECC approach within the organisation
Identifying Resources	Outlines what resources and support are available to assist with this
Infrastructure	Relates to current systems in place to enable MECC to be embedded
Staff Readiness and Engagement	Considers how staff can use their knowledge to promote health and wellbeing
Implementation-training	To increase staff knowledge and skills through MECC training
Review and Evaluation	Refers to monitoring of MECC outcomes to improve delivery

Although MECC aims to be delivered at scale in the United Kingdom, a low awareness has been shown amongst healthcare professionals [16]. Despite healthcare professionals stating that patients would benefit from MECC behaviour change interventions, they were only able to deliver MECC interventions to half of the patients during consultations [16].

Haighton et al. [9] proposed that theoretically relevant behaviour change components should be introduced to interventions to improve MECC implementation. One way of doing this is through introducing a wider range of BCTs which are used as a method for ‘specifying, evaluating, and implementing behaviour change interventions’ (BCTTv1) that link with theoretical domains framework domains. The Theoretical Domains Framework was developed for implementation research to identify theories relevant to implementation and group constructs from these theories into domains [1]. They can be used to address barriers to MECC implementation for example including incentives through use of reward based digital tools for recording MECC [9]. Pre- and post-evaluations of MECC training using the Theoretical Domains Framework have shown that beliefs about capabilities, goals, and staff confidence in delivering behaviour change conversations increased following training [15]. These findings were consistent at follow-up (six to ten weeks post training) showing maintained improvements for healthcare staff delivering MECC.

To evaluate whether interventions are being delivered as intended, intervention fidelity assessments are made [8]. As well as intervention fidelity, treatment fidelity [2], is assessed using five domains: study design, provider training, treatment delivery, treatment receipt and treatment enactment [3]. Intervention fidelity assessments have been used in healthcare contexts such as the NHS diabetes prevention programme [11] to ensure that behavioural intervention programmes are reliable and valid. Fidelity of training programmes has previously been examined in health contexts assessing healthcare professionals’ delivery and treatment provision of specific interventions [12, 21]. However, in some contexts such as the voluntary and community sector fidelity can be difficult to examine due to challenges reporting MECC as there is a lack of standardised resources and monitoring systems and tools that can be externally accessed and used to record and monitor MECC conversations [10].

To assess the fidelity of MECC training, Lawrence et al. [18] examined how trainers adhered to the outlined methods of training. Evidence of fidelity was shown through the interaction of trainers and trainees to model key skills of training and enable trainees to be involved in training activities to increase knowledge and competence. Healthcare staff who have completed MECC training were found to adopt behaviour change approaches with service users and implement MECC skills more readily than non-trained staff [18]. Healthcare professionals consider MECC as an enabler to implementing behaviour change. However, it was also noted that there were inconsistencies in the training approach used across organisations [18]. Therefore, a more streamlined approach to training is needed to ensure consistent delivery of the MECC approach [4, 22].

*Train the trainer* models have been widely used in public health [24, 33], and have been found effective, which is valued by participants [24]. Using *train the trainer* models for public health allows a wide reach of training [33] through a process of trainers delivering training to staff who are then equipped with the skills and knowledge to enable the delivery of cascade training [6]. However, although *train the trainer* models are effective in increasing knowledge amongst healthcare professionals, cascading this training to other staff once trained has shown to be less effective [27]. For example, although *train the trainer* models in public health demonstrated efficiency, when training is then cascaded, fidelity assessments have found low adherence to the original programme content i.e., trainers deviate from planned/original training content [6]. More recently, online training has been developed in Northwest England to support healthcare staff to deliver MECC and increase knowledge of behaviour change techniques [5, 15]. After receiving online MECC training, healthcare staff showed an increase in self-efficacy, behavioural attitudes and outcome expectancies demonstrating that receiving MECC training boosted staff skills in supporting behaviour change [5].

In a mental health inpatient setting in Northern England a training package has been developed to improve staff confidence and skills to ensure consistent delivery of MECC across the trust. The training package includes the *train the trainer* model which involves the delivery of the MECC approach and how to cascade this training amongst peers. A bespoke training was also developed to include Core MECC training with the addition of A Weight Off Your Mind. A Weight Off Your Mind is a regional weight management plan developed in Northeast England to support people living with learning disabilities or severe mental illness with weight management. A Weight Off Your Mind focuses on Physical Activity and Diet to support service users of mental health settings achieve a healthy weight which can be facilitated through the delivery of MECC conversations. The bespoke MECC training adopts the ‘3As approach’ to brief interventions (‘Ask’; ‘Assist’; ‘Act’) often used in smoking cessation interventions [25] and which is adapted from the 5As approach [32] (Ask, Assess, Advise, Agree, Assist). The 5 A’s approach [32] is also an approach used to improve weight management in primary care and shortened to adhere to the brief element of MECC.

To evaluate the MECC training programme, a fidelity assessment was conducted. Intervention fidelity assesses the extent an intervention was delivered as intended [8]. A recent behavioural analysis of MECC interventions [9] highlighted that a low percentage of behaviour change techniques (BCTs) were used across national interventions designed to improve MECC implementation, indicating a potential lack of fidelity of design of MECC, and highlighting a need for interventions to include more behaviour change techniques to overcome barriers to MECC implementation.

### Research aims

This study aimed to assess intervention fidelity in the design of MECC implementation within a mental health inpatient setting. Specifically, a document analysis was conducted to compare MECC provider materials with official guidance documents and a MECC behavioural analysis [9], to determine whether MECC delivery aligned with its intended design. Additionally, the study evaluated training fidelity within the MECC training package by examining whether both components - *Train the trainer* and Core MECC + A Weight Off Your Mind - were being delivered according to the training materials, including presentation slides and the core MECC training delivery guide.

### Research objectives

The objectives of this study were:

1. To examine fidelity of design of MECC implementation across a mental health inpatient setting. This objective was achieved by comparing the behaviour change techniques (BCTs) and implementation strategies outlined in provider and guidance MECC documents.
2. To assess fidelity of training across the same mental health inpatient setting. This was done by analysing BCTs from training session transcripts and comparing them with training documents, including presentation slides and a training delivery guide. The analysis focused on the delivery of both *train the trainer* and Core MECC + A Weight Off Your Mind components.

## Methods

### Fidelity of design

#### Setting

This project took place in a mental health inpatient setting in Northern England.

### Document review

A total of six documents were provided from a current member of staff working in the mental health inpatient setting and part of the wider research team. Documents included MECC Logic Model, MECC Fact Sheet, MECC Evaluation Framework, MECC Plan, implementation Checklist and MECC Implementation Guide. All documents were adapted specifically for the mental health inpatient setting.

- MECC Logic Model, Trust Version: Logic Model developed by mental health inpatient setting, document outlines process and impact of MECC strategy, covering inputs, outputs and outcomes.
- MECC Fact Sheet: A guidance document developed by Health Education England/Maudsley as part of a MECC implementation toolkit.
- MECC Evaluation Framework: A guidance document developed by Health Education England/Maudsley as part of a MECC implementation toolkit.
- MECC Plan: Original plan developed by a Public Health and Wellbeing Lead.
- Implementation Checklist: Developed by a Public Health and Wellbeing Lead.
- MECC Implementation Guide: A guidance document developed by Health Education England/Maudsley as part of a MECC implementation toolkit.

### Coding framework and analysis

A document review was used to identify MECC intervention components/theoretical underpinnings. All materials provided by the mental health inpatient setting were coded for BCTs using the BCTTv1. The Expert Recommendations for Implementing Change (ERIC) Taxonomy [28] was used to identify implementation strategies outlined in the provider documents. The Template for Intervention Description and Replication (TiDieR) checklist [14], a guide to reporting interventions, was used to describe key features of the MECC training, including mode of delivery, who delivered it, where, and at what dose (e.g., duration and frequency). Once identified, BCTs in the MECC implementation documents were then compared to two guidance documents and a recent behavioural analysis of nationally available MECC training packages [9] to assess the fidelity of the design of MECC training across the mental health inpatient setting.

To ensure reliability of coding procedures, all documents were coded initially by one of the authors (EK). Two other authors (AR, MA) then independently coded 50% of the documents each, thus ensuring that 100% of the documents were double coded by two team members. Authors who completed document coding, held meetings to discuss coding discrepancies and ensured agreement was met on all coding before being included in final analysis.

### Fidelity of training

#### Design

A fidelity assessment of MECC training was conducted to assess if the training delivered to staff across the mental health inpatient setting was being performed as intended. Training documents were shared with the research team directly from the mental health inpatient setting and were coded for the presence of different BCTs. Training recordings of two sessions delivered at the trust were shared with the research team and analysed for BCTs. Both coding of training documents and transcripts of recordings were assessed to determine if the MECC training had been delivered as intended.

#### Setting

Training sessions took place via Microsoft Teams or in locations across the mental health inpatient setting during September 2022- March 2023. Training sessions were open to internal staff members working at the mental health inpatient setting who were able to sign up to training via an internal virtual bulletin board.

#### Procedure

Two training sessions (one *train the trainer* December 2022, one Core MECC + A Weight Off Your Mind February 2023) out of a total of five core MECC + A Weight Off Your Mind, and five *train the trainer* sessions between September 2023 and March 2023 were recorded. The two recorded online sessions were observed by one of the authors (EK). Recordings were transcribed and coded for behaviour change techniques (BCTs) content.

#### Materials

Training documents received from the trust included:

- PowerPoint slides of *train the trainer* and Core MECC + A Weight Off Your Mind training with added facilitator notes.
- Core training delivery guide: document explaining how to deliver MECC cascade training.

#### Analysis

All training documents and transcripts of recordings were coded for behaviour change techniques (BCTs) using the BCTTv1. To ensure reliability checks for coding, one author (EK) coded all documents and recording transcripts and a second author (AR) performed 10% coding checks across all documents and recording transcripts. Both authors met to discuss any coding discrepancies and ensured agreement was reached for all codes before presenting final analysis outcomes.

## Results

### Fidelity of design

Table 2 reports the Template for Intervention Description and Replication (TIDieR) checklist to provide an overview of MECC implementation across the mental health inpatient setting and outlines the aims of the training package including *train the trainer* and a bespoke training designed additionally to incorporate the A Weight Off Your Mind strategy.

**Table 2:** Template for intervention description and replication (TIDieR) for the implementation of MECC across the trust

TIDieR Checklist Item	Mental Health Inpatient Setting MECC Implementation
Name	Making Every Contact Count (MECC) implementation
Why (Identified from documents: MECC Logic Model, MECC Fact Sheet, MECC Evaluation Framework, MECC Plan Implementation Checklist, MECC Implementation Guide)	The primary aim of the MECC training programme in the trust is to: • Improve staff confidence in having opportunistic healthy lifestyle conversations with service users. • To deliver healthy lifestyle messages and encourage service users to change their behaviour. • Supporting service users to make positive changes to their physical and mental health. • Increase number of MECC conversations that staff are having with service users about healthy weight, physical activity and healthy eating. • To highlight considerations for the evaluation of MECC programmes that are specific to mental health settings.
What	Materials: Pre- and post-training questionnaires were sent to all participants of the training to enable internal training evaluation. All training resources and copies of training slides are added to the MECC hub website. Procedure: A training package was designed by the trust to deliver two training pathways. The first was a *train the trainer* session to enable staff to gain the skills needed to deliver cascade MECC training to their colleagues. The second was a bespoke training which combined Core MECC training with the element of A Weight Off Your Mind to provide staff with the skills and information needed to deliver MECC conversations. Prior to September 2022 Core MECC training was delivered without the addition of A Weight Off Your Mind. Training was delivered between both in online and face-to-face formats. Planning and implementation of training through A Weight Off Your Mind steering group.
Who	The training is designed by the public health and wellbeing lead in the trust. *Train the trainer* training is delivered by a wellbeing specialist/regional MECC trainer within the trust. Bespoke training is delivered by a health improvement specialist with the trust. Clinical staff working in mental health inpatient settings across the trust are invited to take part in the training.
How	Online training via Microsoft Teams Face-to-face within hospital settings
Where	Northeast England mental health inpatient setting
When and how much	Post COVID-19, the training package relaunched in September 2022. The initial planned sessions occurred between September-December 2022, then recommenced in March 2023 and are currently ongoing. *Train the trainer* training sessions lasted 3 h, and Core MECC + A Weight Off Your Mind were 90 min. 5 *train the trainer* and 15 core MECC training sessions have been delivered since September 2022.
Tailoring	Both training sessions included showing staff examples of MECC scripts for A Weight Off Your Mind following the 3 A’s structure which focused on healthy weight management, physical activity and alcohol consumption to prepare staff how to deliver MECC conversations.
Modifications	No modifications
How well	No intervention adherence or fidelity assessed

### Behaviour Change Techniques (BCTs) content

A total of 31 unique BCTs (Table 3) were identified across the provider and guidance documents. The largest number of identified BCTs in the provider documents were found in the MECC Evaluation Framework which included 16 BCTs. The MECC Fact Sheet contained five BCTs, the MECC Implementation Guide included four BCTs and the MECC Implementation Checklist included six BCTs. Of the guidance documents, most BCTs were identified in the guidance MECC Implementation Guide (*n* = 11), followed by eight in the behavioural analysis and four in the guidance MECC Implementation Checklist (Table 3).

**Table 3:**
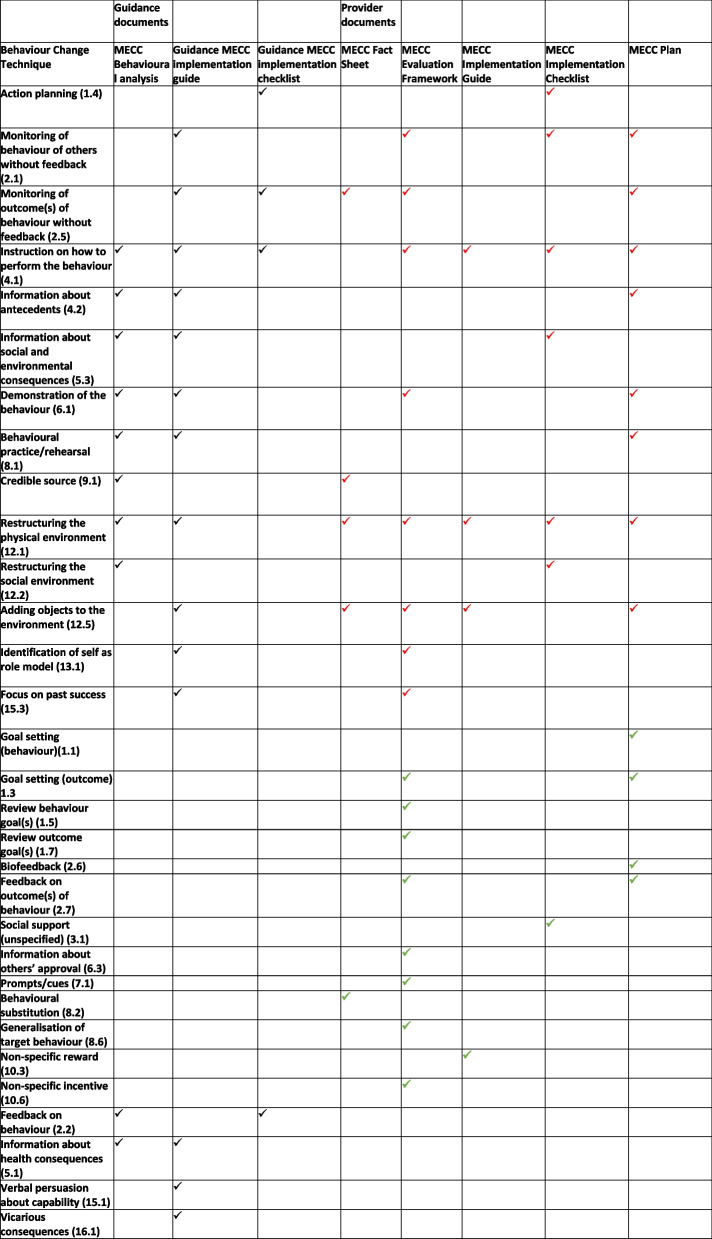
Identified BCTs from the Behaviour Change Technique taxonomy (v1) for guidance and provider documents

Numbers in brackets correspond to BCTs identified from Behaviour Change Technique Taxonomy (v1)

Black ticks refer to all BCTs identified from guidance documents which should be present in provider documents

Red ticks refer to BCTs that were identified in both guidance and provider documents

Green ticks refer to BCTs that were identified in provider documents but not present in guidance documents

### Expert Recommendations for Implementing Change (ERIC) strategies

Twenty-six implementation strategies were identified overall (Table 4). Six implementation strategies appeared in the MECC Logic Model, seven in the MECC Evaluation Framework, four in the MECC Plan and 12 in the MECC Implementation Guide (see Table 4).

**Table 4:**
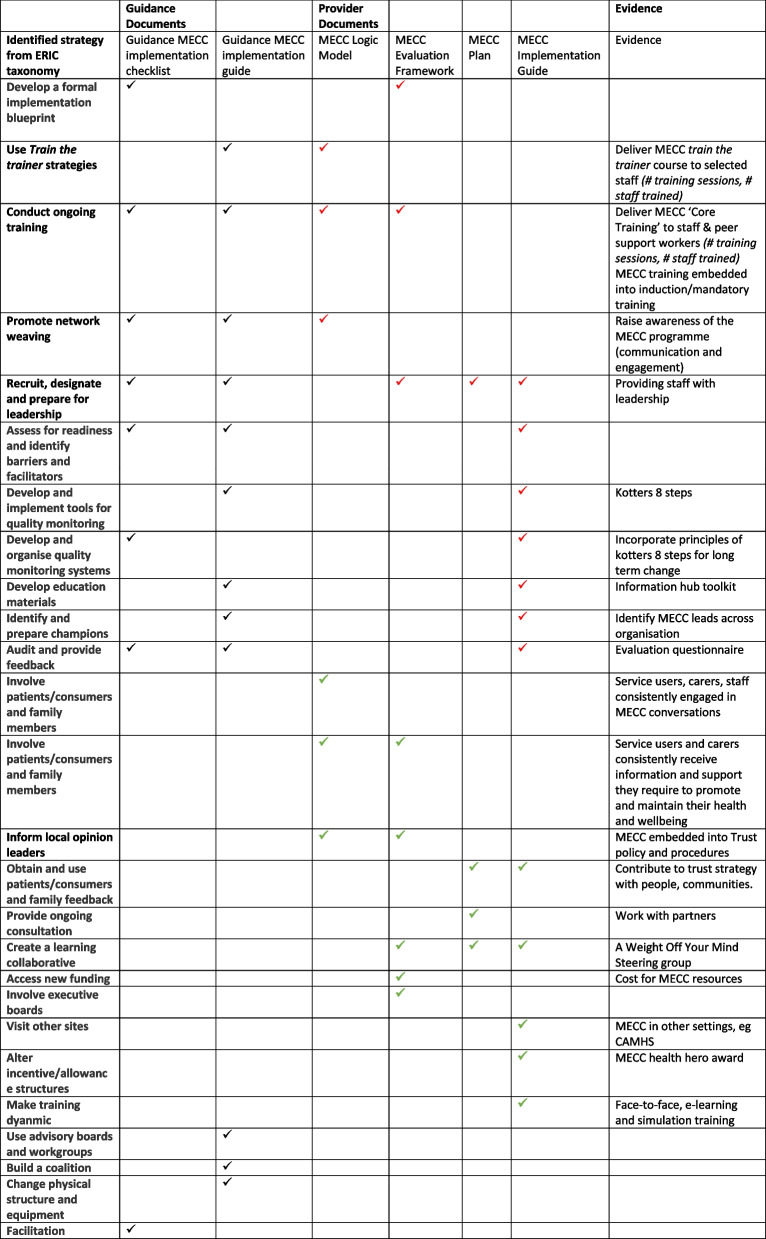
Identified implementation strategies from the Expert Recommendations for Implementing Change (ERIC) Taxonomy

Black ticks refer to all ERIC strategies identified from guidance documents which should be present in provider documents

Red ticks refer to ERIC strategies that were identified in both guidance and provider documents

Green ticks refer to ERIC strategies that were identified in provider documents but not present in guidance documents

### Mapping identified BCTs to guidance documents and behavioural analysis

Table 3 shows which BCTs were present across provider and guidance MECC documents and the MECC behavioural analysis [9]. It is evident that of the 18 BCTs included in the guidance documents and behavioural analysis 14 (77%) appeared in the provider documents. Following guidance on fidelity assessment [19] 77% indicates overall moderate fidelity (80–100% = high fidelity, < 50% =low fidelity) of adherence from provider MECC documents to guidance documents. The BCTs missing included ‘Feedback on behaviour’ (2.2), ‘Information about health consequences’ (5.1), ‘Verbal persuasion about capability’ (15.1) and ‘Vicarious consequences’ (16.1). Following guidance on fidelity assessment [19] 77% indicates overall moderate fidelity (80–100% = high fidelity, < 50% =low fidelity) of adherence from provider MECC documents to guidance documents. The analysis of the provider documents also found an additional 13 (42%) BCTs to those present in the guidance documents. This could suggest a loss of fidelity to MECC guidance, as these extra BCTs were not part of the original framework.

Across the provider and guidance documents, the most commonly occurring BCT was ‘Instruction on how to perform the behaviour’ (4.1). ‘Restructuring the physical environment’ (12.1) was identified in the MECC implementation guidance and MECC behavioural analysis [9] and appeared across all documents from the mental health inpatient setting. From the 15 BCTs identified in the guidance MECC implementation guide, 13 (86%) appeared across the mental health inpatient setting documents (the BCTs ‘Information about health consequences’ (5.1) and ‘Verbal persuasion about capability’ (15.1) were omitted from the BCTs present in the mental health inpatient setting documents). Of the four BCTs identified in the MECC implementation checklist guidance, two (50%) appeared in the mental health inpatient setting documents.

A total of 26 implementation strategies were identified from both provider and guidance documents with 8 appearing in the MECC implementation checklist guidance and 12 in the MECC implementation guidance. Table 4 shows that from the 16 implementation strategies identified across the guidance documents and behavioural analysis, 4 were absent from the provider documents, which included, ‘Use advisory boards and workgroups’, ‘Change physical structure and equipment’, ‘Build a coalition’ and ‘Facilitation’. This shows high fidelity [19] of the implementation of MECC in the mental health inpatient setting with an 81% adherence of strategies from the provider documents when compared to the guidance documents and behavioural analysis [9]. However, a loss of fidelity through additional Expert Recommendations for Implementing Change (ERIC) strategies was also seen, as the provider documents included an additional 11 implementation strategies showing that out of 26 identified strategies overall, 42% were additional strategies identified from the provider documents showing potential loss of fidelity.

### Assessing fidelity of identified BCTs from training materials to training transcripts

Throughout the analysis process it became apparent that most BCTs were targeted towards staff delivering both MECC conversations and MECC cascade training however some BCTs related to service users receiving MECC therefore Table 5 shows BCTs applied to both staff and service users. From the Core MECC + A Weight Off Your Mind transcript, 15 BCTs were recorded that directly related to staff and 12 BCTs were focused on service users. From the *train the trainer* transcript, 25 BCTs were recorded as being aimed towards staff and seven were patient focused. Analysis of the training recording transcripts showed that all planned BCTs from the training documents were delivered during either the bespoke Core MECC + A Weight Off Your Mind or *train the trainer* sessions.

**Table 5:**
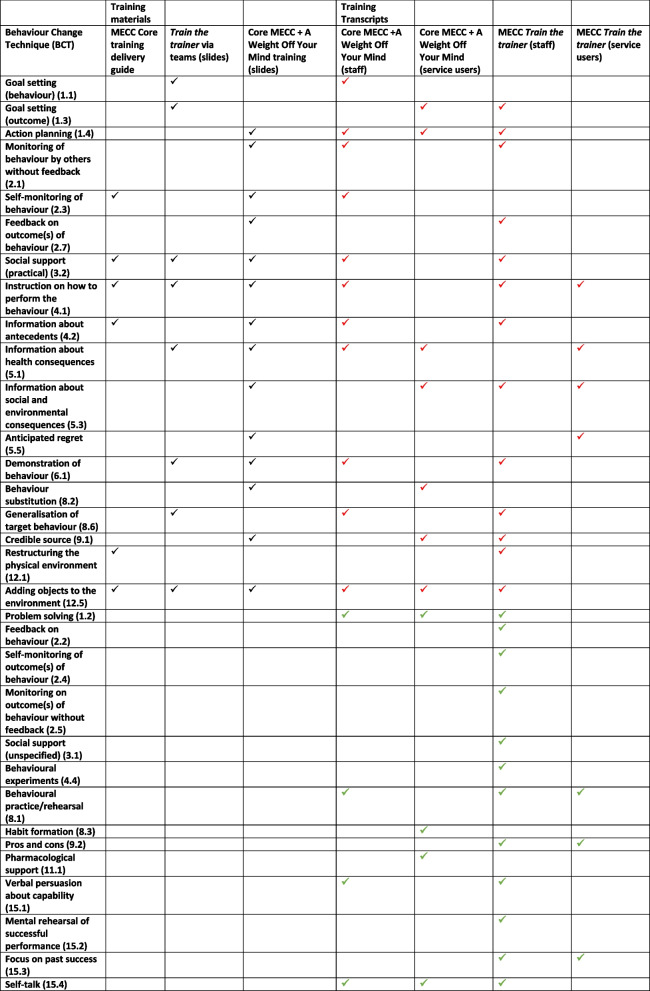
Identified BCTs from training materials and training transcripts

Black ticks refer to all BCTS identified from training materials which should be present in training transcripts

Red ticks refer to BCTs that were identified in both training materials and transcripts

Green ticks refer to BCTs identified only in training transcripts

### Fidelity of training

Overall, 32 unique BCTs were identified from the training materials shared by the mental health inpatient setting and the training transcripts for core MECC + A Weight Off Your Mind and *train the trainer* training sessions. Of the 18 BCTs identified from the training materials, 100% were also identified in the training transcripts, indicating high fidelity [19] of adherence to delivering MECC training to training materials including presenter slides and training guide. However, in addition to the 18 BCTs identified from the training materials, 14 additional BCTs were identified, showing that during the training sessions the content of the training documents was expanded upon indicating a possible loss of fidelity through additional behaviour change techniques. Core MECC + A Weight Off Your Mind training contained 14 BCTs, *train the trainer* training contained 8 BCTs, and the core MECC training delivery guide contained 6 identified BCTs. The BCTs which appeared across all documents were ‘Social support (practical) (3.2)’, ‘Instruction on how to perform the behaviour (4.1)’, and ‘Adding objects to the environment (12.5)’. Table 6 shows the list of most common BCTs staff were trained in across both training sessions (appearing in both sessions).

**Table 6:** Key BCTs identified from training recordings

BCT	Present in training documents
Problem solving (1.2)	**X**
Action planning (1.4)	✔
Instruction on how to perform the behaviour (4.1)	✔
Information about health consequences (5.1)	✔
Information about social and environmental consequences (5.3)	✔
Demonstration of the behaviour (6.1)	✔
Behavioural practice/rehearsal (8.1)	**X**
Pros and Cons (9.2)	**X**
Adding objects to the environment (12.5)	✔
Self-talk (15.4)	**X**

Ten out of 32 identified BCTs from the training transcripts appeared to be key BCTs occurring most frequently throughout the training or across both sessions. Four or 40% of those BCTs were unplanned and not present in the analysis of the training materials including training delivery guide and presentation slides.

## Discussion

From evaluating both fidelity of design and fidelity of staff training across the mental health inpatient setting, it was apparent that the service demonstrated good fidelity to the guidance for delivering MECC training across the trust. The highest fidelity of design applied to the MECC Implementation Guide showing that MECC was being delivered as intended from a guidance to provider perspective. Implementation of the MECC training package across the mental health inpatient setting demonstrated good fidelity to the training delivery guide and showed that content delivered during the training sessions was reflective of what appeared in the training slides with additional content added throughout the session. A potential loss of fidelity could be seen through additional unplanned BCTs and implementation strategies. The analysis found 42% additional BCTs, and 42% additional Expert Recommendations for Implementing Change (ERIC) strategies in provider documents when compared to guidance documents.

Previous research has shown that *train the trainer* models are effective in increasing knowledge amongst healthcare staff [18, 27] which could reflect the findings of the current study, as high fidelity of training was shown when comparing the training content delivered to staff which is designed to improve knowledge. The training fidelity assessment found that overall, training transcripts showed high fidelity (100%) to the training documents which included guidance and presenter slides, however the training also provided additional BCTs not identified from the materials which could indicate a loss of fidelity. Although tailoring to suit individual populations may occur, for example applying MECC training to mental health settings, adaptations should be kept to a minimum to ensure adherence to initial programs [7]. However, appropriate adaptation may occur specific to context [31], therefore, this training may have adapted to additional factors such as further audience participation in the training sessions and responses from audience leading to further BCTs being implemented during the online training sessions. Lawrence et al. [18] found that evidence of fidelity was shown through trainer and trainee interactions during training sessions which led to increased staff knowledge and competence, which could explain why additional BCTs were present during the training sessions. As previous research has identified lack of training to be a barrier to delivery of MECC [9], our findings provide evidence of the fidelity of MECC training delivered to healthcare staff in a mental health inpatient setting. This evidence could potentially inform further implementation of MECC training across other regions throughout the UK.

### Research and practice implications

This study is the first to examine the fidelity of design and training in a mental health inpatient setting, and to specifically assess a newly developed bespoke training package which incorporates core MECC training with the element of A Weight Off Your Mind. Previous research has shown that 80–100% adherence indicates high fidelity and less than 50% indicates low fidelity [19]. The current study showed that overall, the mental health inpatient setting was delivering moderate fidelity of design and high fidelity of training. Moderate fidelity was seen across the document analysis showing that the implementation of MECC across the mental health inpatient setting was adhering to MECC guidance. High fidelity was seen across MECC training programme and appeared to be successfully delivered as planned in the training delivery guide and training materials.

The lowest fidelity score resulted from the MECC implementation guidance when compared to the documents provided from the mental health inpatient setting - only a 50% BCT match was present, indicating low fidelity to the implementation checklist. It appeared that ‘Action planning (1.4)’ was present in the guidance MECC implementation checklist however this did not appear in the provider documents provided by the mental health inpatient setting. This could be a useful inclusion in the provider documents to highlight areas of planning to ensure MECC is being implemented as intended based on guidance. The second BCT ‘Feedback on behaviour’ (2.2) was identified from the guidance MECC checklist however it did not appear in the provider documents. This may also be a useful BCT to include in the mental health inpatient setting as this could ensure successful monitoring and feedback of the implementation of MECC and allow the trust to adapt based on feedback. However, both highlighted techniques are self-regulatory techniques that have been tested extensively in various settings (e.g., feedback, action planning, self-monitoring) [20] and these discrepancies could also be due to the mental health inpatient setting having designed their own implementation checklist which is adapted from the national guide but specific to the region.

The findings from this study have the potential to lead to beneficial outcomes for public health and across the trust, as previous research has shown that lack of awareness amongst staff can lead to fewer opportunistic conversations between staff and service users [16]. Delivering training to staff as intended by the training delivery guide and materials can lead to increased awareness of MECC across staff and enable staff to gain the skills and confidence required to deliver opportunistic conversations to service users at appropriate times and have knowledge of signposting and resources to encourage healthy lifestyle behaviour change. This has been reported previously where staff who received MECC training used greater skills and supported behaviour change in service users more than non-trained staff [18].

### Strengths and limitations

This study has provided an evaluation of the training programme currently being implemented in a mental health inpatient setting that aims to promote MECC training through a *train the trainer* model enabling staff to cascade MECC training to colleagues and a bespoke MECC training which combines the core elements of MECC with A Weight Off Your Mind which has a specific focus on promoting healthy weight management. The fidelity analysis has provided a good understanding of how MECC training is currently being delivered in the mental health inpatient setting and the main BCTs being targeted to encourage successful implementation of MECC delivered to service users across the trust. However, there are limitations to consider, for example the findings of this study are reflective of one mental health inpatient setting based in one region of the UK, therefore are not reflective of the wider scale of MECC implementation.

The Template for Intervention Description and Replication (TIDieR)checklist (Table 2) identified that currently no specific intervention adherence or fidelity checks are in place to assess adherence of MECC implementation in the mental health inpatient setting to national guidance. To formally examine fidelity in the trust a fidelity assessment would be recommended, this could lead to improved outcomes for training evaluation as a training session could be monitored to assess if it is being delivered regionally as intended in accordance with MECC guidance. Despite the fidelity evaluation producing encouraging findings regarding MECC training across the mental health inpatient setting, the recordings were of training which took place online only. As the trust had designed training to be delivered in both online and face-to-face settings it would have been useful to also evaluate face-to-face training to examine if the BCTs were being delivered to staff or if additional BCTs were included during the session due to increased audience participation and discussion.

The aim of the *train the trainer* model was to enable staff to gain the skills needed to provide cascade MECC training to their colleagues to embed MECC across the trust and increase number of MECC trained staff. A further limitation of this study was that cascade training was not examined; therefore, it was not possible to assess fidelity of delivery of MECC training to see if the planned BCTs during *train the trainer* sessions are being delivered as intended in subsequent cascade training sessions. This would be a useful further step in the research to examine if fidelity of training is consistent throughout cascade training session. This would lead to increased awareness of implementation of MECC across the mental health inpatient setting and an understanding of how effective cascade training sessions are from staff who have participated in *train the trainer* MECC training.

## Conclusion

Overall, this fidelity analysis demonstrated that the mental health inpatient setting was delivering moderate fidelity of design and high fidelity of training. The implementation of MECC in the mental health inpatient setting to promote opportunistic health lifestyle brief interventions during routine consultations was shown to have potential to lead to positive outcomes from the trust as the training was being delivered as intended. This could impact staff confidence and enable skills and knowledge to be increased and ability to deliver successful brief interventions via healthy lifestyle conversations during routine contact with the service to enable MECC to be embedded across the mental health inpatient setting. Further research into the *train the trainer* model is needed to assess the effectiveness of the cascade element and to assess fidelity of delivery of cascade training in a public health focused initiative (MECC). The current fidelity analysis found that in the mental health inpatient setting, *train the trainer* model appears to be implemented as intended demonstrating high fidelity, however there are limits to assessing fidelity in a mental health inpatient setting due to patient capacity and time constraints to partake in cascade training.

## Data Availability

The datasets used and/or analysed during the current study are available from the corresponding author on reasonable request.
